# Supplementary material for: Myoglobin-loaded gadolinium nanotexaphyrins for oxygen synergy and imaging-guided radiosensitization therapy
Source: Nat Commun. 2023 Oct 4;14:6187. doi: 10.1038/s41467-023-41782-w (PMC10550994; doi:10.1038/s41467-023-41782-w)
Supplement: Supplementary file 1 — Supplementary Information File [file 41467_2023_41782_MOESM1_ESM.pdf]

Supporting Information

Title: Myoglobin-Loaded Gadolinium Nanotexaphyrins for Oxygen Synergy and Imaging-Guided Radiosensitization Therapy

Authors: *Xiaotu Ma[1,2], Xiaolong Liang[2], Meinan Yao[3], Yu Gao[1], Qi Luo[4], Xiaoda Li[5], Yue Yu[1], Yining Sun[1], Miffy H. Y. Cheng[6], Juan Chen[6], Gang Zheng[6,7]*, Jiyun Shi[1]*, and Fan Wang[1,3,4]**

Affiliations:

[1]Key Laboratory of Biomacromolecules, CAS Center for Excellence in Biomacromolecules, Institute of Biophysics, Chinese Academy of Sciences, Beijing, 100101, China

[2]Department of Ultrasound, Peking University Third Hospital; Beijing 100191, P. R. China.

[3]Medical Isotopes Research Center and Department of Radiation Medicine, State Key Laboratory of Natural and Biomimetic Drugs, School of Basic Medical Sciences, International Cancer Institute, Peking University; Beijing 100191, P. R. China.

[4]Guangzhou National Laboratory; Guangzhou 510005, P.R. China.

[5]Medical and Health Analysis Center, Peking University; Beijing 100191, P. R. China.

[6]Princess Margaret Cancer Centre, University Health Network, Tronto; Ontario M5G 1L7, Canada.

[7]Department of Medical Biophysics, University of Toronto; Ontario M5G 1L7 Canada.

*Corresponding author. E-mail: gang.zheng@uhnres.utoronto.ca (Gang Zheng); shijiyun@ibp.ac.cn (Jiyun Shi); wangfan@bjmu.edu.cn (Fan Wang).

Supplementary Table 1. Pharmacokinetic parameters of Cy5.5-labeled agents.

Parameter*	Unit	Mb	Hb	Mb@NTs	Mb@Gd-NTs	Hb@NTs	Hb@Gd-NTs
$t_{1/2\alpha}$	h	0.12	0.42	0.62	0.82	0.78	0.76
$t_{1/2\beta}$	h	2.96	4.18	25.67	23.98	30.31	29.63
**CL1**	(%ID)/(%ID/mL)/h	19.52	12.18	0.034	0.041	0.026	0.029
**CL2**	(%ID)/(%ID/mL)/h	52.80	6.07	0.31	0.26	0.21	0.26
$AUC_{0\text{-}48\ h}$	%ID/mL*h	5.12	23.07	2178.13	1897.62	2538.41	2328.93
**MRT**	h	3.45	4.63	36.57	33.82	43.29	42.10

* $t_{1/2\alpha}$, half-live of the distribution phase; $t_{1/2\beta}$, half-live of the elimination phase; CL, clearance rate; $AUC_{0\text{-}48\ h}$, area under the curve from 0 h to 48 h; MRT, mean residence time.

Supplementary Table 2. Statistical analysis of Fig. 5d. Statistical analysis was performed by a two-tailed unpaired *t* test. *, $P < 0.05$; **, $P < 0.01$; ***, $P < 0.001$.

	G1	G2	G3	G4	G5	G6	G7	G8
**G2**	*P = 0.0244							
**G3**	**P = 0.0018	**P = 0.0064						
**G4**	**P = 0.0018	**P = 0.0018	**P = 0.0018					
**G5**	**P = 0.0018	**P = 0.0018	**P = 0.0018	P = 0.1642				
**G6**	**P = 0.0018	**P = 0.0018	**P = 0.0018	P = 0.0830	P = 0.9505			
**G7**	**P = 0.0018	**P = 0.0018	**P = 0.0018	P = 0.5739	P = 0.2917	P = 0.1546		
**G8**	**P = 0.0018	**P = 0.0018	**P = 0.0018	**P = 0.0018	**P = 0.0064	**P = 0.0064	**P = 0.0018	
**G9**	**P = 0.0018	**P = 0.0018	**P = 0.0018	**P = 0.0018	**P = 0.0018	**P = 0.0018	**P = 0.0018	*P = 0.0494

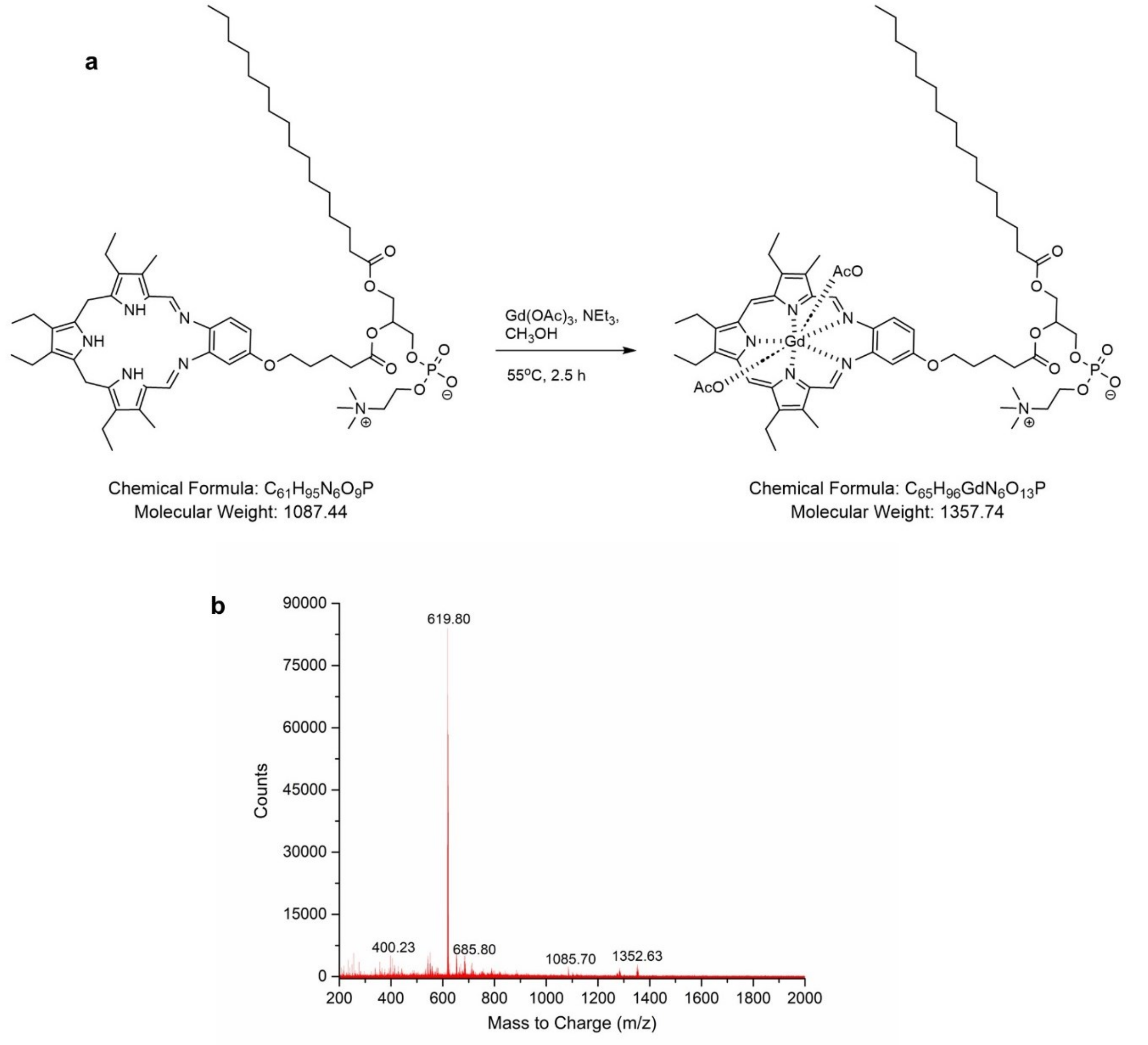

Supplementary Figure 1. Synthesis and verification of Gd-Tex-lipid. (a) Synthesis of Gd-Tex-lipid. **(b)** ESI-Q-TOF mass spectrum of Gd-Tex-lipid. Theoretical molecular weight of Gd-Tex-lipid coordinated with two acetates (AcO^-): 1357.74. The peak of m/z was found at 619.80 ($[M-2AcO]^{2+}$). The experiment (b) was repeated three times independently with similar results.

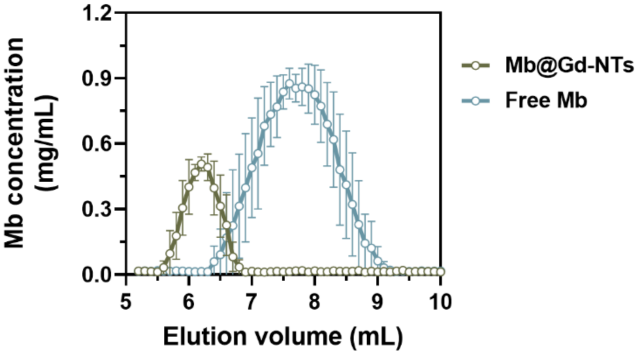

Supplementary Figure 2. Examination of unencapsulated Mb in Mb@Gd-NTs by size exclusion chromatography. The possible unencapsulated Mb (globular protein, ~ 17 kDa) in Mb@Gd-NTs was examined using the gel exclusion column containing Sephadex G-100 with a fractionation range of 4000-150000 Da for globular proteins. Free Mb or Mb@Gd-NTs was added to the columns, and the protein concentration of eluent was measured using BCA (bicinchoninic acid) Protein Assay Kit. Free Mb was eluted in volume fractions between 6.3 and 9.2 ml with the peak at 7.6 mL, but Mb@Gd-NTs (purified by dialysis) were quickly eluted in volume fractions between 5.5 and 6.9 ml with the peak at 6.2 mL, which showed only one peak in the chromatogram, demonstrating that the unencapsulated Mb can be almost completely removed by the dialysis bag with a molecular cutoff of 1000 kDa. The data are shown as the mean ± SD (n = 3 independent experiments). Source data are provided as a Source Data file.

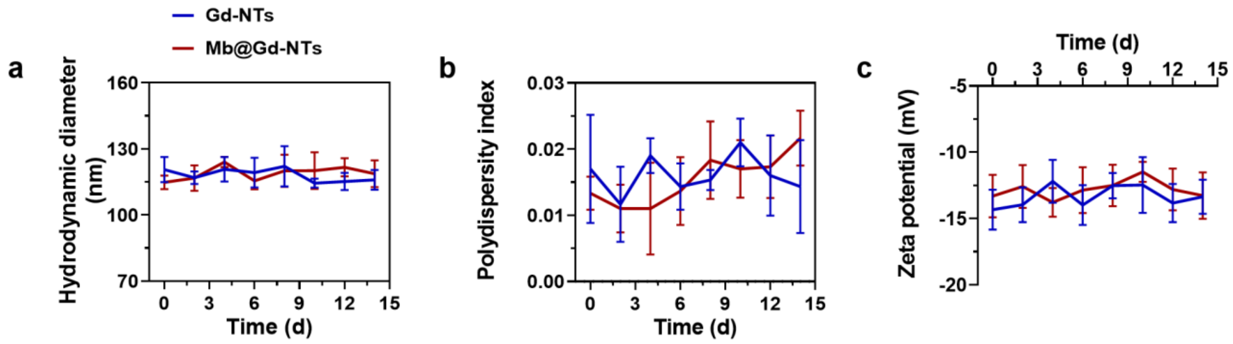

Supplementary Figure 3. Stability of Gd-NTs and Mb@Gd-NTs stored at 4 °C for 14 days, as assessed by changes in hydrodynamic diameter (a), polydispersity index (b) and zeta potential (c). The data (a-c) are shown as the mean ± SD (n = 3 independent experiments). Source data are provided as a Source Data file.

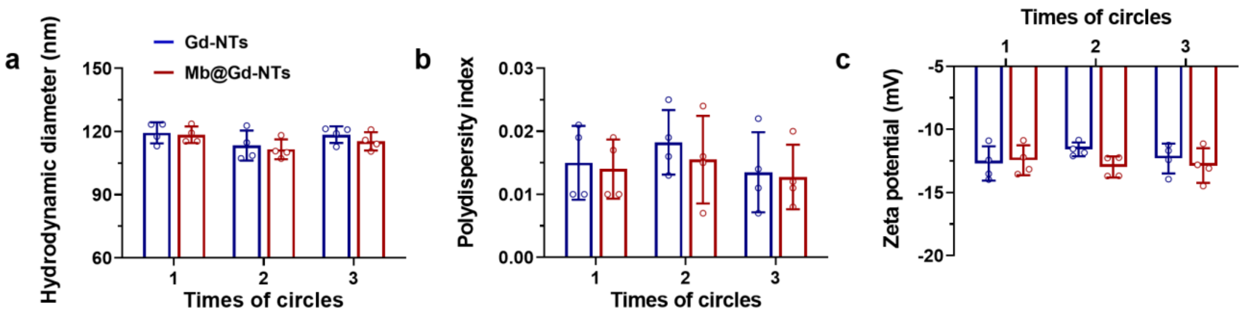

Supplementary Figure 4. Stability of Gd-NTs and Mb@Gd-NTs after three freeze–thaw cycles, as assessed by changes in hydrodynamic diameter (a), polydispersity index (b) and zeta potential (c). The data (a-c) are shown as the mean ± SD (n = 4 independent samples). Source data are provided as a Source Data file.

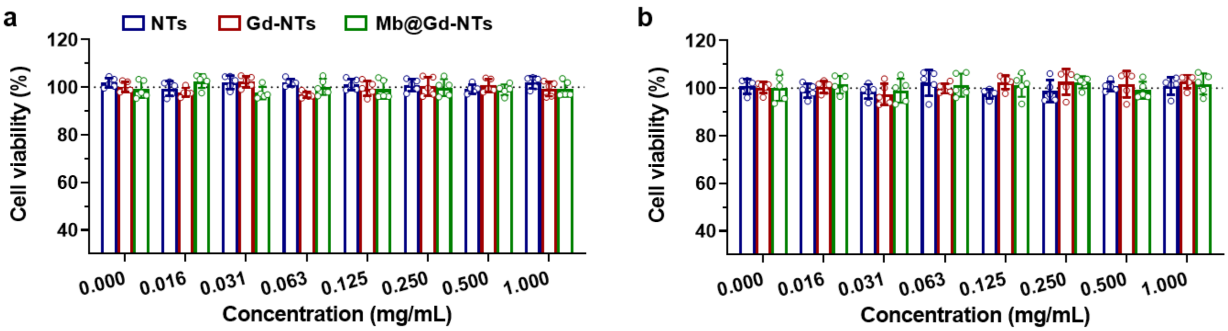

Supplementary Figure 5. Cell viability after incubating cells with different concentrations of NTs, Gd-NTs, and Mb@Gd-NTs, exhibiting good biocompatibility. The concentration was calculated as the total amount of lipids. (a) Human umbilical vein vessel endothelial cells, HUVECs; (b) 293T human embryonic kidney cells. The data (a-b) are shown as the mean ± SD (n = 5 independent samples). Source data are provided as a Source Data file.

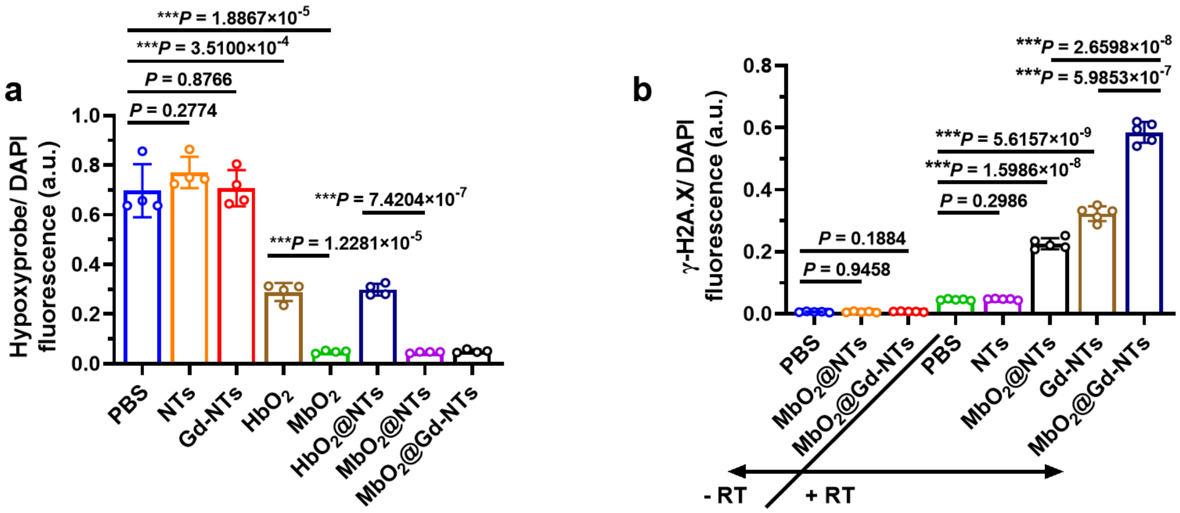

Supplementary Figure 6. Semiquantification results of Figure 2a (a) and Figure 2d (b), exhibiting the cellular hypoxia level (a) and DNA DSB level (b) of LLC cells after incubating cells with various agents under hypoxic conditions. The data are shown as the mean ± SD (n = 4 independent samples for panel a; n = 5 independent samples for panel b). Statistical analysis was performed by a two-tailed unpaired *t* test. *, $P < 0.05$; **, $P < 0.01$; ***, $P < 0.001$. Source data are provided as a Source Data file.

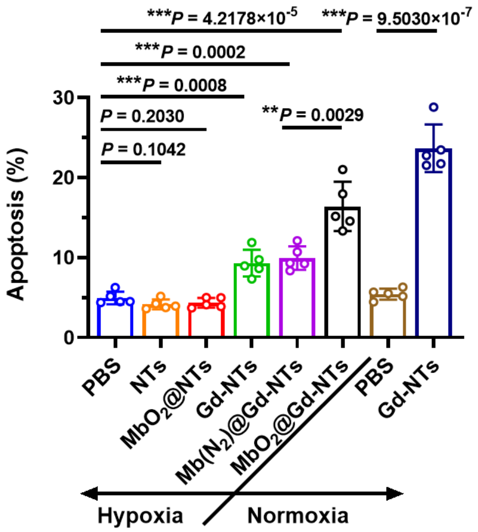

Supplementary Figure 7. Quantification results of Figure 2e, exhibiting cellular apoptosis after incubating cells with various agents under hypoxic or normoxic conditions and treating cells without X-ray irradiation. The sum of Annexin V-positive and PI-positive cells in total cells was calculated as the percentage of apoptotic cells. The data are shown as the mean ± SD (n = 5 independent samples). Statistical analysis was performed by a two-tailed unpaired *t* test. *, $P < 0.05$; **, $P < 0.01$; ***, $P < 0.001$. Source data are provided as a Source Data file.

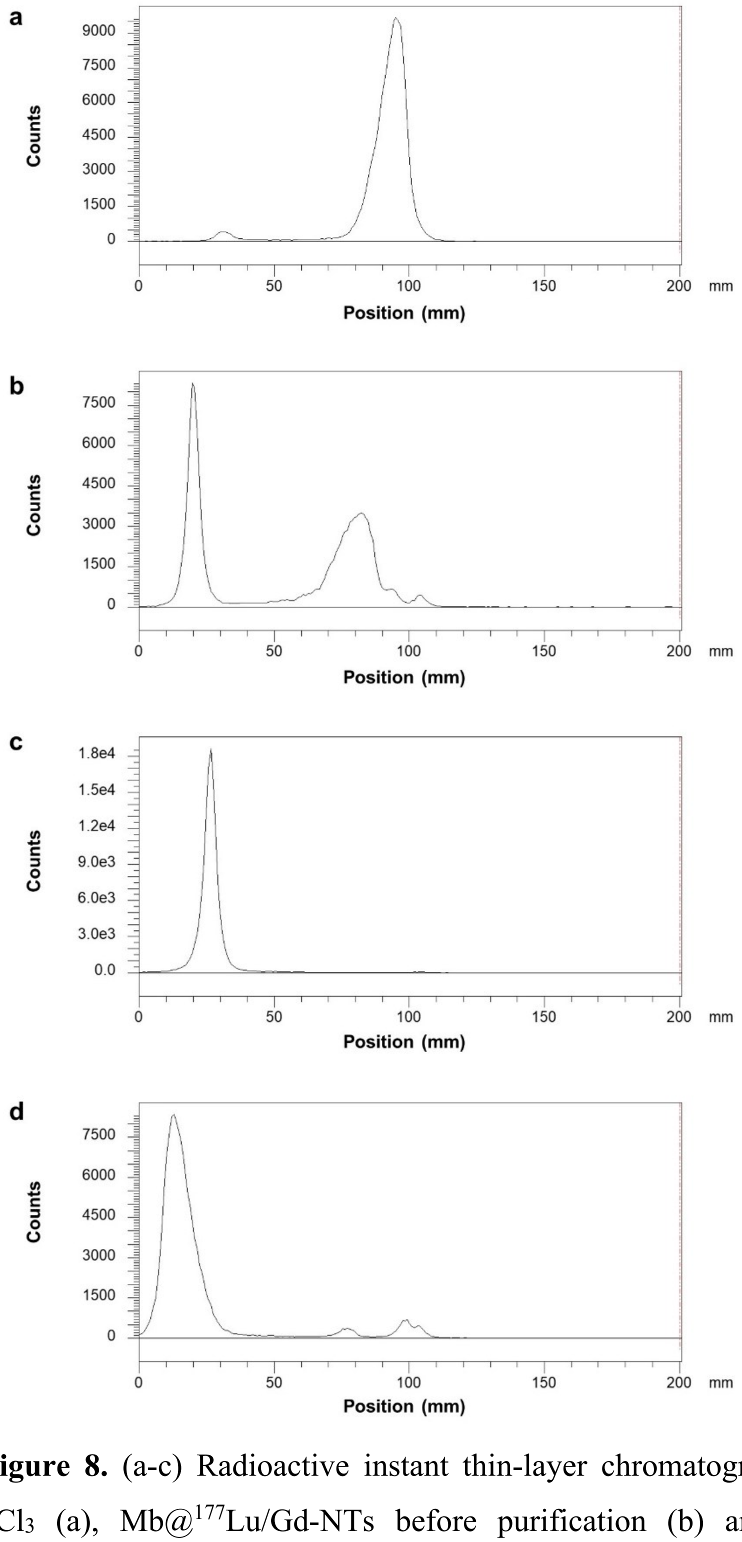

Supplementary Figure 8. (a-c) Radioactive instant thin-layer chromatography (Radio-ITLC) analysis of $^{177}LuCl_3$ (a), Mb@^{177}Lu/Gd-NTs before purification (b) and ^{177}Lu-NTs after purification (c). The radiochemical purity of Mb@^{177}Lu/Gd-NTs was > 98% after purification. (d) Radio-ITLC analysis of Mb@^{177}Lu/Gd-NTs dissociated with 1% Triton X-100. Only a small

portion of $^{177}Lu^{3+}$ (< 10%) was encapsulated into the hydrophilic core of liposomes rather than forming ^{177}Lu-Tex-lipid. These experiments (a-d) were repeated three times independently with similar results.

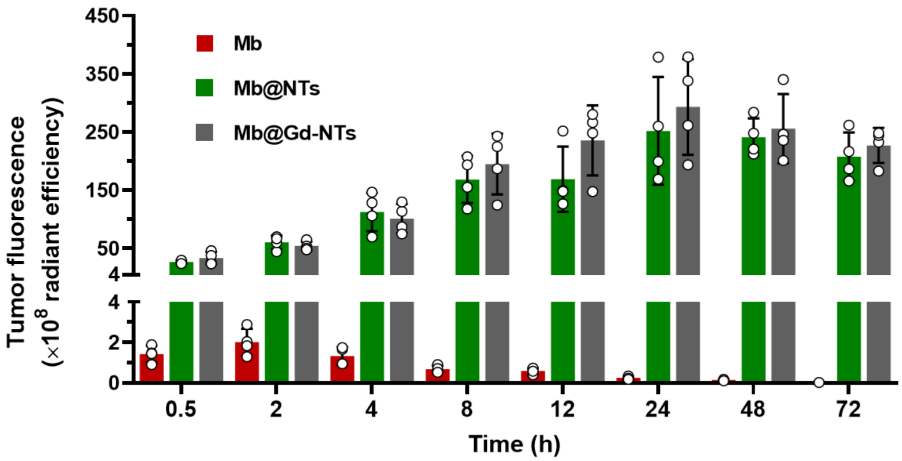

Supplementary Figure 9. Semiquantitative analysis of the fluorescence intensity of the tumor region in Figure 3f. The data are shown as the mean ± SD (n = 4 mice). Source data are provided as a Source Data file.

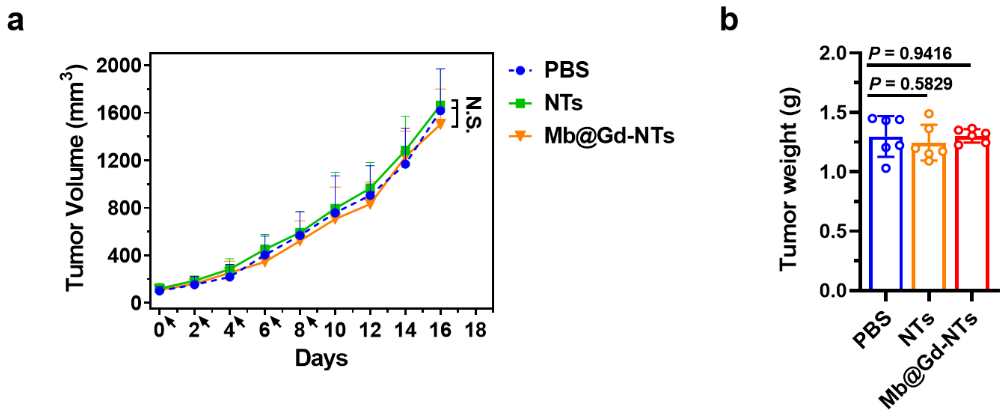

Supplementary Figure 10. (a) The change in tumor volume after five rounds of RT indicated by the black arrows. (b) The tumor weight of different treatment groups weighed on day 16. The tumor growth curve and tumor weight of PBS were copied from Figure 4b-c for comparison. The data are shown as the mean ± SD (n = 6 mice). Statistical analysis was performed by a two-tailed unpaired *t* test. *, $P < 0.05$; **, $P < 0.01$; ***, $P < 0.001$; N.S. (no siginificance), $P > 0.05$. Source data are provided as a Source Data file.

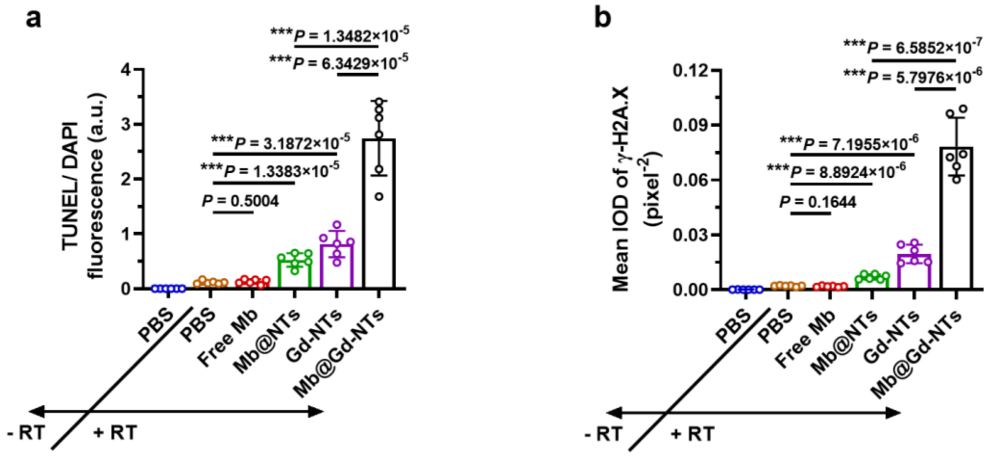

Supplementary Figure 11. (a) Semi-quantification results of Figure 4f, exhibiting the SSB level of tumor tissue sections. (b) Semi-quantification of mean integrated optical density (IOD) of γ-H2A.X in tumor tissue sections of Figure 4g. Mean IOD = (IOD of γ-H2A.X-positive region) / (Total area of tissue). The data (a-b) are shown as the mean ± SD (n = 6 mice). Statistical analysis was performed by a two-tailed unpaired *t* test. *, $P < 0.05$; **, $P < 0.01$; ***, $P < 0.001$. Source data are provided as a Source Data file.

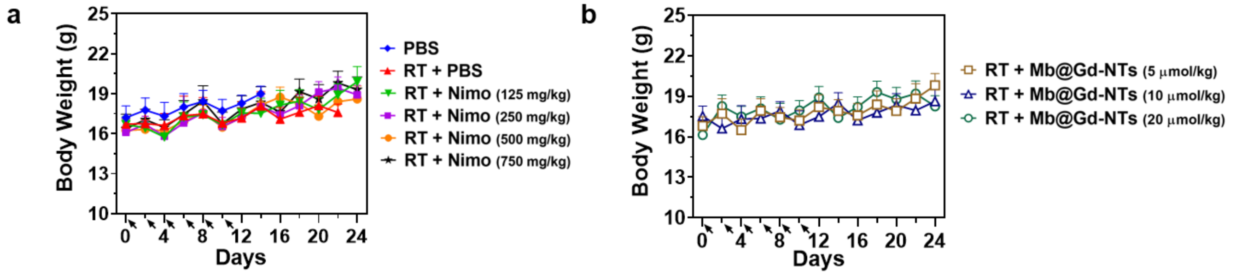

Supplementary Figure 12. The change of body weight of mice measured every two days. LLC tumor-bearing mice were treated with six sessions of RT on days 0, 2, 4, 6, 8, and 10. Different doses of nimorazole (500-750 mg/kg) were i.p. injected 30 min before each RT session (a), and different doses of Mb@Gd-NTs (5-20 µmol/kg) were i.v. injected 24 h before each RT session (b). The data (a-b) are shown as the mean ± SD (n = 5 mice). Source data are provided as a Source Data file.

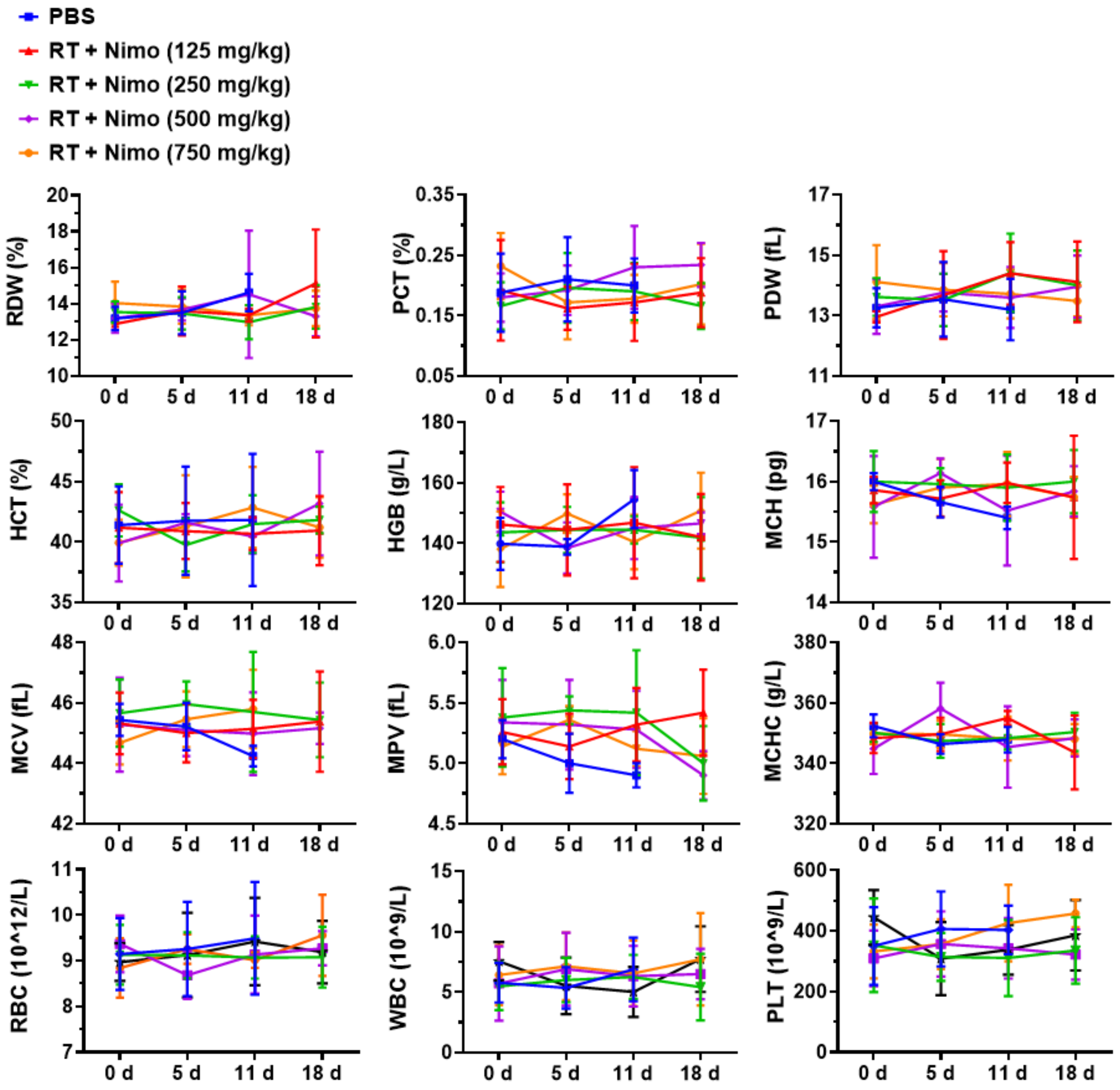

Supplementary Figure 13. Blood routine examination performed on days 0, 5, 11, 18. LLC tumor-bearing mice were treated with six sessions of RT on days 0, 2, 4, 6, 8, and 10. Different doses of nimorazole (Nimo, 500-750 mg/kg) were i.p. injected 30 min before each RT session. The data are shown as the mean ± SD (n = 5 mice). Source data are provided as a Source Data file.

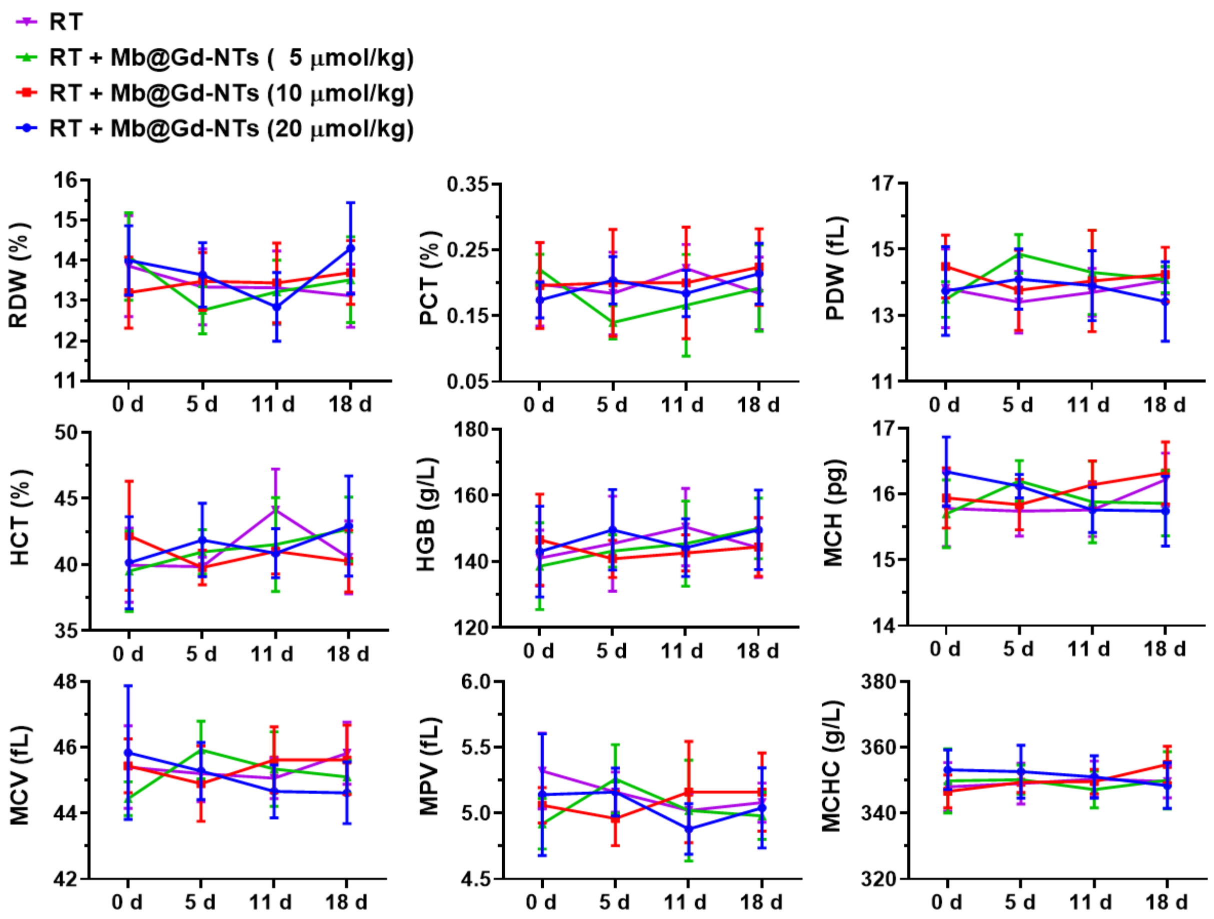

Supplementary Figure 14. Blood routine examination performed on days 0, 5, 11, 18. LLC tumor-bearing mice were treated with six sessions of RT on days 0, 2, 4, 6, 8, and 10. Different doses of Mb@Gd-NTs (5-20 μmol/kg) were i.v. injected 24 h before each RT session. The data are shown as the mean ± SD (n = 5 mice). Source data are provided as a Source Data file.

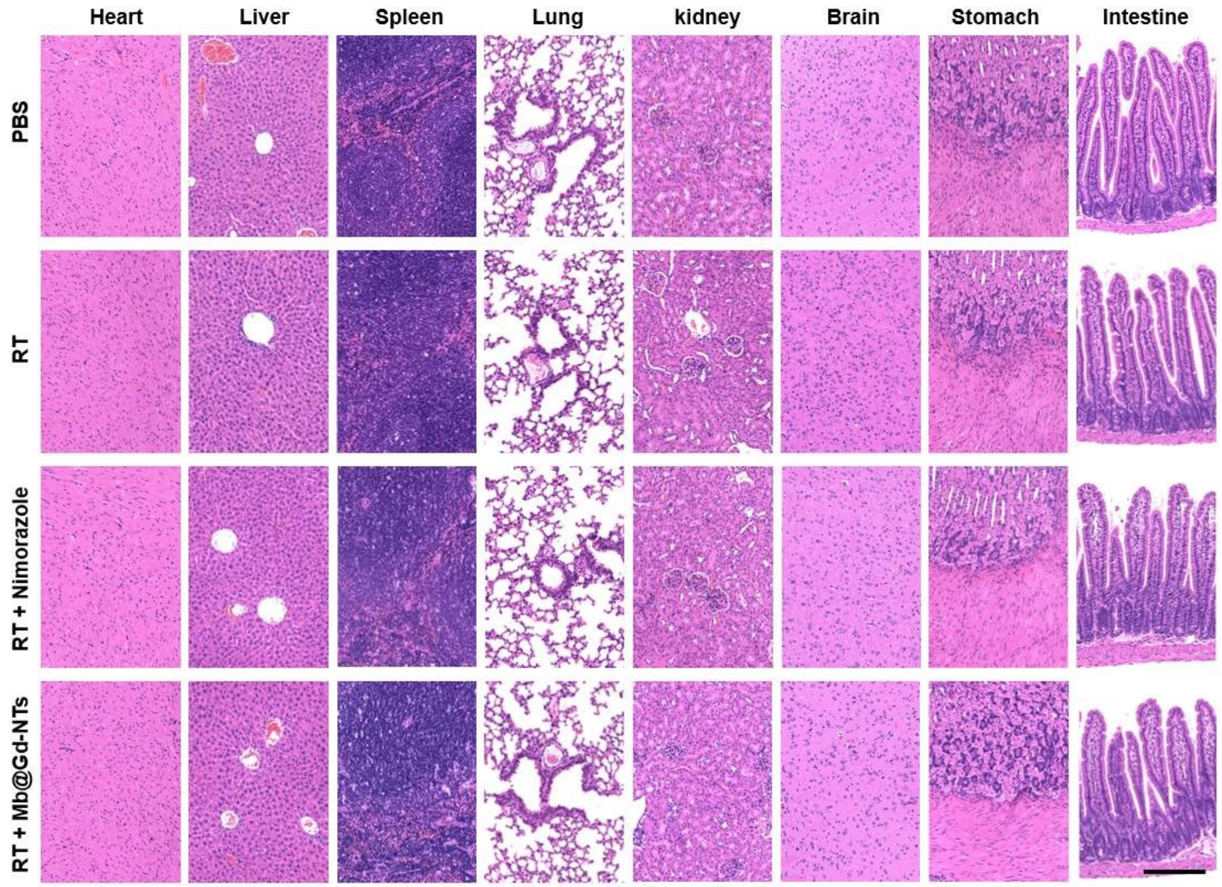

Supplementary Figure 15. Histological examination of normal organs performed on day 24. LLC tumor-bearing mice were treated with six sessions of RT on days 0, 2, 4, 6, 8, and 10. Mb@Gd-NTs (20μmol/kg) were i.v. injected 24 h before each RT, and nimorazole (750 mg/kg) was i.p. injected 30 min before each RT session. Scale bar, 200 μm. These experiments were repeated three times independently with similar results.

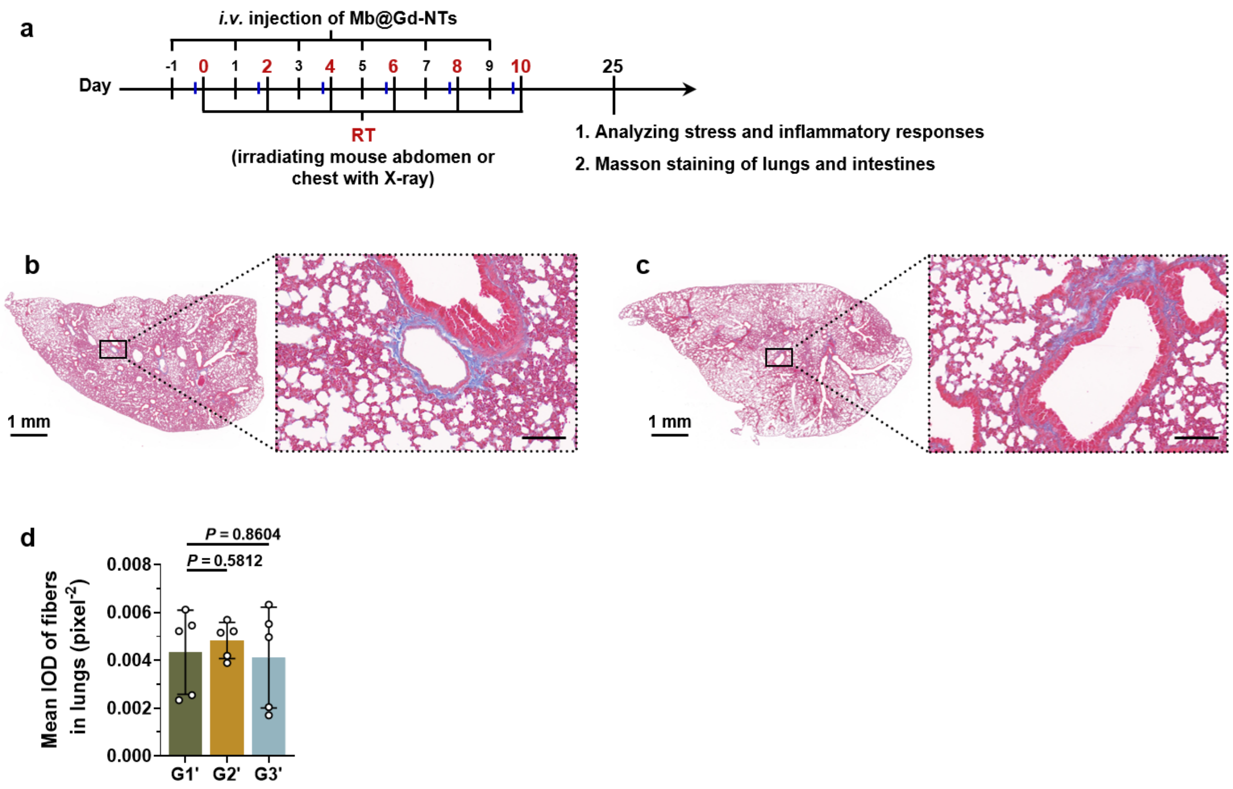

Supplementary Figure 16. The examination of radiation-induced fibrosis of lungs. (a) The schedule of examining radiation-induced side effects of the intestine and lung. 6-week-old C57BL/6 mice were divided into three groups (n = 5) with different treatments: (G1') PBS; (G2') PBS + RT; (G3') Mb@Gd-NTs + RT. For evaluating side effects on the intestine or lung, mouse abdomen or chest was respectively irradiated with a dose of 2 Gy on days 0, 2, 4, 6, 8, 10 for a total dose of 12 Gy. Mb@Gd-NTs were i.v. injected with a dose of 27.15 mg/kg (20 μmol/kg) 24 h before each RT session. Mice were sacrificed for necropsy on day 25, and the intestines and lungs were collected for the analysis of oxidation stress and inflammatory responses and Masson staining of tissue sections. **(b-c)** Masson staining of lung sections of G1' (b) and G2' (c) showing the distribution of collagen in lungs (n = 5 mice). Cells and nucleus were stained red and dark blue with ponceau and hematoxylin, while collagen fibers were stained blue with aniline blue. Scale bar for the right panels, 100 μm. These experiments (a-c) were repeated three times independently with similar results. **(d)** Semi-quantification of mean integrated optical density (IOD) of fiber in lung sections. Mean IOD = (IOD of fiber-positive region) / (Total area of tissue). The data (d) are shown as the mean ± SD (n = 5 mice). Statistical analysis was performed by a two-tailed unpaired *t* test. *, $P < 0.05$; **, $P < 0.01$; ***, $P < 0.001$. Source data are provided as a Source Data file.

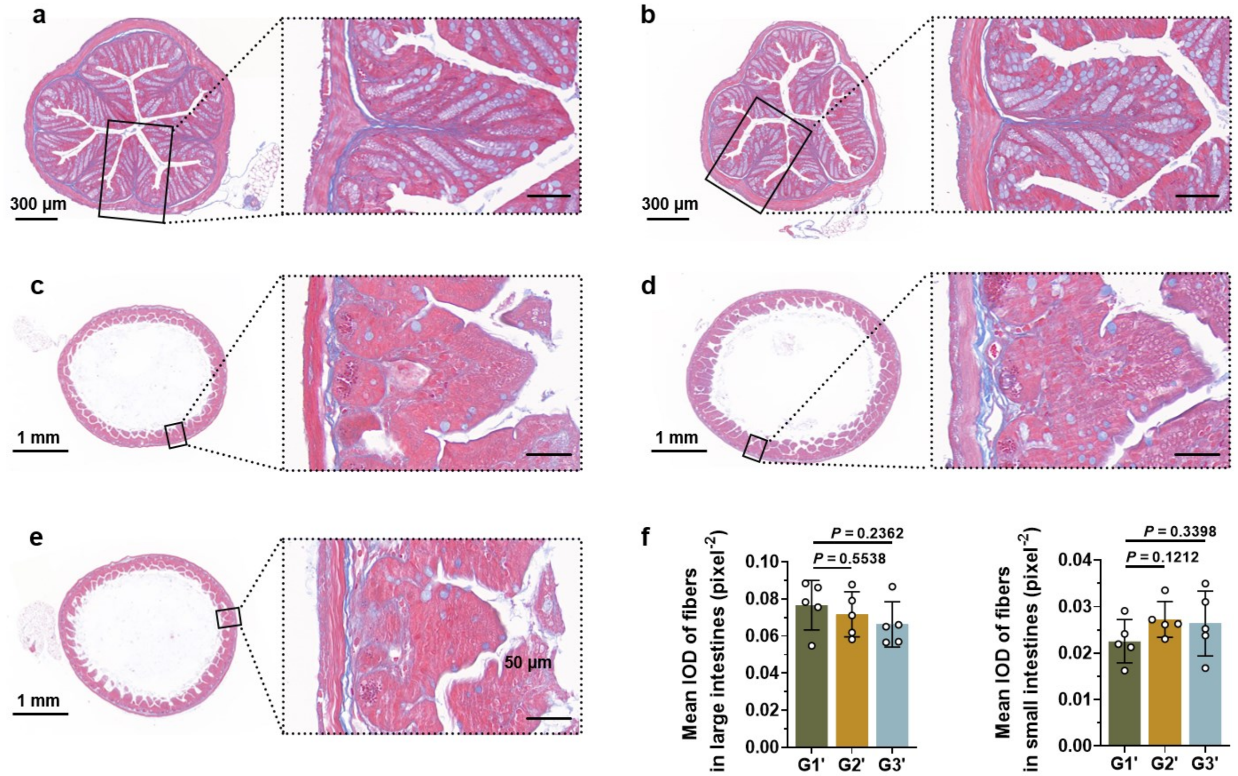

Supplementary Figure 17. The examination of radiation-induced fibrosis of intestines. (a-b) Masson staining of colorectum sections of G1’ (a) and G2’ (b) showing the distribution of collagen in colorectum (n = 5 mice). **(c-e)** Masson staining of small intestine sections of G1’ (c), G2’ (d), and G3’ (e) showing the distribution of collagen in small intestines (n = 5 mice). Cells and nucleus were stained red and dark blue with ponceau and hematoxylin, while collagen fibers were stained blue with aniline blue. Scale bar for the enlarged views, 100 μm. These experiments (a-e) were repeated three times independently with similar results. **(f)** Semi-quantification of mean integrated optical density (IOD) of fibers in large and small intestine sections. The data (f) are shown as the mean ± SD (n = 5 mice). Statistical analysis was performed by a two-tailed unpaired *t* test. *, $P < 0.05$; **, $P < 0.01$; ***, $P < 0.001$. Source data are provided as a Source Data file.

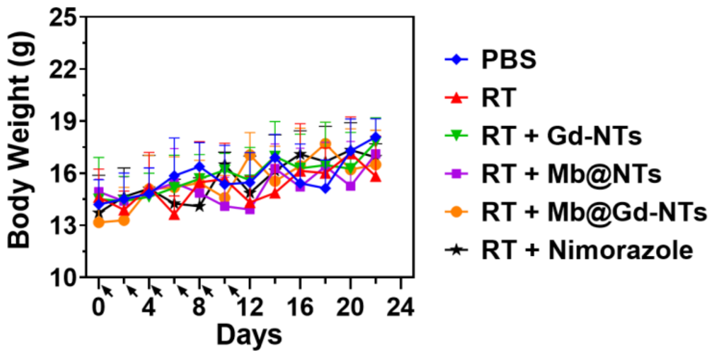

Supplementary Figure 18. The change of body weight of mice measured every two days. MCF-7 tumor-bearing mice were treated with six sessions of RT on days 0, 2, 4, 6, 8, and 10. Gd-NTs, Mb@NTs, and Mb@Gd-NTs at a dose of 20 μmol/kg were i.v. injected 24 h before each RT session, and nimorazole at a dose of 750 mg/kg was i.p. injected 30 min before each RT session. The data are shown as the mean ± SD (n = 5 mice). Source data are provided as a Source Data file.

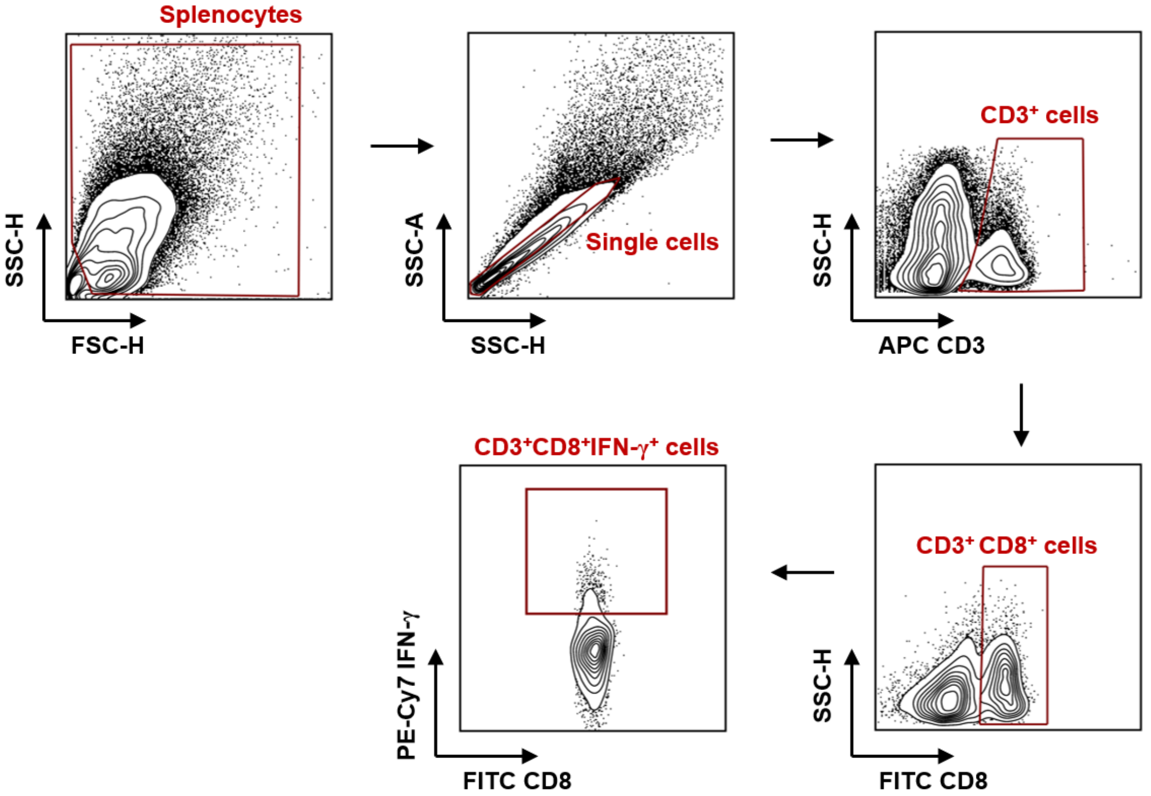

Supplementary Figure 19. Gating strategy for CD3^{+}CD8^{+}IFN-γ^{+} cells in splenocytes after re-stimulation with whole cell lysate of LLC cells (cf. Figure 7d-e).
