# Supplementary material for: Myoglobin-loaded gadolinium nanotexaphyrins for oxygen synergy and imaging-guided radiosensitization therapy
Source: Nat Commun. 2023 Oct 4;14:6187. doi: 10.1038/s41467-023-41782-w (PMC10550994; doi:10.1038/s41467-023-41782-w)
Supplement: Supplementary file 3 — Reporting Summary [file 41467_2023_41782_MOESM3_ESM.pdf]

nature portfolio

Corresponding author(s): Gang Zheng, Jiyun Shi, Fan Wang

Last updated by author(s): Aug 16, 2023

Reporting Summary

Nature Portfolio wishes to improve the reproducibility of the work that we publish. This form provides structure for consistency and transparency in reporting. For further information on Nature Portfolio policies, see our Editorial Policies and the Editorial Policy Checklist.

Statistics

For all statistical analyses, confirm that the following items are present in the figure legend, table legend, main text, or Methods section.

n/a	Confirmed	
☐	☒	The exact sample size (*n*) for each experimental group/condition, given as a discrete number and unit of measurement
☐	☒	A statement on whether measurements were taken from distinct samples or whether the same sample was measured repeatedly
☐	☒	The statistical test(s) used AND whether they are one- or two-sided<br>*Only common tests should be described solely by name; describe more complex techniques in the Methods section.*
☒	☐	A description of all covariates tested
☐	☒	A description of any assumptions or corrections, such as tests of normality and adjustment for multiple comparisons
☐	☒	A full description of the statistical parameters including central tendency (e.g. means) or other basic estimates (e.g. regression coefficient) AND variation (e.g. standard deviation) or associated estimates of uncertainty (e.g. confidence intervals)
☐	☒	For null hypothesis testing, the test statistic (e.g. *F*, *t*, *r*) with confidence intervals, effect sizes, degrees of freedom and *P* value noted<br>*Give P values as exact values whenever suitable.*
☒	☐	For Bayesian analysis, information on the choice of priors and Markov chain Monte Carlo settings
☒	☐	For hierarchical and complex designs, identification of the appropriate level for tests and full reporting of outcomes
☒	☐	Estimates of effect sizes (e.g. Cohen's *d*, Pearson's *r*), indicating how they were calculated

Our web collection on statistics for biologists contains articles on many of the points above.

Software and code

Policy information about availability of computer code

Data collection	NovoExpress (version number, 1.4.1.1901) and BD FACSCalibur Software (version number, 1.0.264.21) were used to collect data of flow cytometry. Living Image software (version number, 4.3.1.16427) was used for the bioluminescence and fluorescence imaging. ZEN 2012 (version number, 1.1.13346.204) was used for the immunofluorescence detection.
Data analysis	ImageJ (version number, 1.8.0) was used for the semi-quantification of immunofluorescence images. FlowJo (version number, 10.0.0.0), NovoExpress (version number, 1.4.1.1901) and BD FACSCalibur Software (version number, 1.0.264.21) were used to analyze the data of flow cytometry. GraphPad Prism (version number, 8.3.0.538) and IBM SPSS Statistics (version number, 19.0) were used for the statistical analysis.

For manuscripts utilizing custom algorithms or software that are central to the research but not yet described in published literature, software must be made available to editors and reviewers. We strongly encourage code deposition in a community repository (e.g. GitHub). See the Nature Portfolio guidelines for submitting code & software for further information.

Data

Policy information about availability of data

All manuscripts must include a data availability statement. This statement should provide the following information, where applicable:

- Accession codes, unique identifiers, or web links for publicly available datasets
- A description of any restrictions on data availability
- For clinical datasets or third party data, please ensure that the statement adheres to our policy

The authors declare that all the data supporting the results in this study are available within the Article, Supplementary Information or Source Data file. The gating strategy for flow cytometry experiments can be found in Supplementary Figure 19. Source data are provided with this paper.

Research involving human participants, their data, or biological material

Policy information about studies with human participants or human data. See also policy information about sex, gender (identity/presentation), and sexual orientation and race, ethnicity and racism.

Reporting on sex and gender	Not applicable.
Reporting on race, ethnicity, or other socially relevant groupings	Not applicable.
Population characteristics	Not applicable.
Recruitment	Not applicable.
Ethics oversight	Not applicable.

Note that full information on the approval of the study protocol must also be provided in the manuscript.

Field-specific reporting

Please select the one below that is the best fit for your research. If you are not sure, read the appropriate sections before making your selection.

☒ Life sciences ☐ Behavioural & social sciences ☐ Ecological, evolutionary & environmental sciences

For a reference copy of the document with all sections, see nature.com/documents/nr-reporting-summary-flat.pdf

Life sciences study design

All studies must disclose on these points even when the disclosure is negative.

Sample size	No statistical methods were used to determine sample size. Sample sizes were determined on the basis of the previous experimental experience (Refs: Nature Communications 2021, 12: 2041; Science Translational Medicine 2021, 13: eabc2816; Nature Biomedical Engineering 2017, 1 : 667-679; Advanced Materials 2021 33: e2006007). In vitro experiments were performed with at least 3 biologically independent samples. All in vivo experiments were performed with at least 3 independent animals. Sample sizes were sufficient to perform statistical analyses.
Data exclusions	No samples were excluded.
Replication	All attempts at replication were successful. Experimental repeat numbers are also reported in Figure Legends.
Randomization	All samples/organisms were numbered and randomly grouped by random number table method.
Blinding	All experimental procedures and quantification of results, including injections, isolation of the tumors or organs, tissue histological analysis and flow cytometry, were done by two independent researchers. Meanwhile, all researchers were blinded to group allocation.

Reporting for specific materials, systems and methods

We require information from authors about some types of materials, experimental systems and methods used in many studies. Here, indicate whether each material, system or method listed is relevant to your study. If you are not sure if a list item applies to your research, read the appropriate section before selecting a response.

Materials & experimental systems

n/a	Involved in the study
☐	☒ Antibodies
☐	☒ Eukaryotic cell lines
☒	☐ Palaeontology and archaeology
☐	☒ Animals and other organisms
☒	☐ Clinical data
☒	☐ Dual use research of concern
☒	☐ Plants

Methods

n/a	Involved in the study
☒	☐ ChIP-seq
☐	☒ Flow cytometry
☒	☐ MRI-based neuroimaging

Antibodies

Antibodies used	Rabbit anti-γ-H2A.X (phospho S139) antibody (Catalog No. ab81299, Clone: EP854(2)Y, 1: 250) was purchased from Abcam (USA). FITC-conjugated goat anti-rabbit IgG H&L antibody (Catalog No. ab6717, 1: 1000) was purchased from Abcam (USA). APC anti-mouse CD3 antibody (Catalog No. 100312, Clone: 145-2C11, 1: 100), FITC anti-mouse CD8 antibody (Catalog No. 100706, Clone: 53-6.7, 1: 100), PE/Cyanine7 anti-mouse IFN-γ antibody (Catalog No. 505826, Clone: XMG1.2, 1: 100), PE anti-mouse/human CD44 antibody (Catalog No. 103008, Clone: IM7, 1: 100), and PE/Cyanine7 anti-mouse CD62L antibody (Catalog No. 104418, Clone: MEL-14, 1: 100) were purchased from BioLegend (USA).
Validation	All antibodies were purchased from the supplier as noted above and used without additional validation. The validation of all the antibodies could be found from manufacturers online:<br>https://www.abcam.cn/gamma-h2ax-phospho-s139-antibody-ep8542y-ab81299.html<br>https://www.abcam.cn/goat-rabbit-igg-hl-fitc-ab6717.html<br>https://www.biolegend.com/en-us/products/apc-anti-mouse-cd3epsilon-antibody-21<br>https://www.biolegend.com/en-us/products/fitc-anti-mouse-cd8a-antibody-153<br>https://www.biolegend.com/en-us/products/pe-cyanine7-anti-mouse-ifn-gamma-antibody-5865<br>https://www.biolegend.com/en-us/products/pe-anti-mouse-human-cd44-antibody-2206<br>https://www.biolegend.com/en-us/products/pe-cyanine7-anti-mouse-cd62l-antibody-1922

Eukaryotic cell lines

Policy information about cell lines and Sex and Gender in Research

Cell line source(s)	The LLC (Catalog No. CRL-1642) and MCF-7 (Catalog No. HTB-22) cell lines was originally obtained from American Type Culture Collection (ATCC; Manassas, VA, USA). The MC38 cell line (resource No. 1101MOU-PUMC000523) was obtained from the Cell Resource Center, Peking Union Medical College (which is the headquarter of National Science & Technology Infrastructure--National BioMedical Cell-Line Resource, NSTI-BMCR, China).Luciferase-expressing MCF-7 (MCF-7 luc+) cells was obtained by stably transfecting MCF-7 cells with the encoding gene of luciferase (Gene ID: 116160065) using FectinMoreTM Transfection Reagent (Catalog No. CM001, Chamot Biotechnology Co., Ltd., China).
Authentication	LLC, MC38 and MCF-7 cells were not performed with authentication.
Mycoplasma contamination	LLC, MC38 and MCF-7 lines were carried out with mycoplasma detection and were negative for mycoplasma contamination using Quick Cell Mycoplasma Assay kit (Catalog No. AC16L061, Life-iLab, China).
Commonly misidentified lines<br>(See ICLAC register)	LLC, MC38 and MCF-7 cell lines are not listed in the database of commonly misidentified lines maintained by the International Cell Line Authentication Committee.

Animals and other research organisms

Policy information about studies involving animals; ARRIVE guidelines recommended for reporting animal research, and Sex and Gender in Research

Laboratory animals	All animal studies were performed in accordance with ARRIVE guidelines. 5-week-old female C57BL/6 mice or BALB/c mice were obtained from Vital River Laboratory Animal Technology Co. Ltd (China). All animal experiments were approved by the Institutional Animal Care and Use Committee at the Institute of Biophysics, Chinese Academy of Science. Mice were housed in a room with a temperature of 20-22 °C and a humidity of 30-70%. Feed and water were available ad libitum. Artificial light was provided in a 12 h light/12 h dark cycle. This study complied with relevant ethical regulations for animal testing and research.
Wild animals	This study did not involve wild animals.
Reporting on sex	All animal experiments were conducted on female mice.
Field-collected samples	The study did not involve samples collected from the field.
Ethics oversight	All animal studies were approved by the Institutional Animal Care and Use Committee at the Institute of Biophysics, Chinese

Ethics oversight | Academy of Science.

Note that full information on the approval of the study protocol must also be provided in the manuscript.

Flow Cytometry

Plots

Confirm that:

☒ The axis labels state the marker and fluorochrome used (e.g. CD4-FITC).

☒ The axis scales are clearly visible. Include numbers along axes only for bottom left plot of group (a 'group' is an analysis of identical markers).

☒ All plots are contour plots with outliers or pseudocolor plots.

☒ A numerical value for number of cells or percentage (with statistics) is provided.

Methodology

Sample preparation

For the detection of cellular ROS and apoptosis, LLC cells were seeded in a 24-well plate at a density of 10^5 cells, cultured at 20% O2 for cell adherence, and further incubated at 1% O2 for 24 h to induce cells to become hypoxic. Cells were incubated with various nanoparticles dissolved in 1 mL of DMEM for 4 h at 1% O2 and repeatedly incubated with the same nanoparticles for 4 h once again. Cells were irradiated with an X-ray irradiator (Rad Source RS2000XE, USA) at a dose of 2 Gy, followed by different assays. (1) Cellular ROS levels were detected using a H2DCFDA probe (Reactive Oxygen Species Assay Kit, Catalog No.O040, LABLEAD Inc., China). After X-ray irradiation, the cells were incubated with 10 µM H2DCFDA for 30 min, digested with 0.05% trypsin (ZOMANBIO, China), and collected to analyze the fluorescence intensity of H2DCFDA using a flow cytometer (BD FACSCalibur, USA). (2) Cell apoptosis was examined using an Annexin V-PI double staining kit (Solarbio, China). The adherent and floating cells were collected 12 h after X-ray irradiation, stained with Annexin V and PI according to the manufacturer's instructions, and analyzed using a flow cytometer.
For analyzing immune memory, the C57BL/6 mice were inoculated with LLC cells (2 X 10^6 cells/mouse) at day -9. For Mb@Gd-NTs + RT, the tumor sites were irradiated with a dose of 2 Gy on days 0, 2, 4, 6, 8, and 10 for a total dose of 12 Gy, and Mb@Gd-NTs were intravenously injected with a dose of 27.15 mg/kg (20 µmol/kg) 24 h before each RT session. For PBS + RT, tumors of mice were irradiated with a dose of 10 Gy on days 0, 2, 4, 6, and 8 for a total dose of 50 Gy, enabling the eradication of tumors. Healthy mice without tumor inoculation (naïve mice) were adopted as controls. Peripheral blood and splenocytes were obtained from mice on day 90. The antigen-specific CD8+ T cells (IFNγ+ cytotoxic T lymphocyte cells) in splenocytes were analyzed by flow cytometry. The memory T cells in peripheral blood and splenocytes were analyzed by flow cytometry, which were categorized as Tnaive (CD3+CD8+CD44-CD62L+), Tcm (CD3+CD8+CD44+CD62L+), and Tem (CD3+CD8 +CD44+CD62L-).

Instrument

Agilent NovoCyte (Agilent Technologies Inc., USA) and BD FACSCalibur (BD Biosciences, USA) were used to analyze samples.

Software

NovoExpress (version number, 1.4.1.1901) and BD FACSCalibur Software (version number, 1.0.264.21) were used to collect data of flow cytometry. FlowJo (version number, 10.0.0.0), NovoExpress (version number, 1.4.1.1901) and BD FACSCalibur Software (version number, 1.0.264.21) were used to analyze the data of flow cytometry.

Cell population abundance

Over 10000 cells were analyzed for fluorescent intensity in the defined gate.

Gating strategy

A gate is drawn around the cells. Single cells are determined with the area and the height of the side scatter (SSC). The analysis was carried out in this gate.

☒ Tick this box to confirm that a figure exemplifying the gating strategy is provided in the Supplementary Information.
